# Giant solitary fibrous tumor of the thoracic cavity presenting with sudden circulatory failure after blunt trauma: a case report

**DOI:** 10.3389/fonc.2026.1832518

**Published:** 2026-05-07

**Authors:** Jun Wang, Shu Cong

**Affiliations:** 1 Department of Intensive Care Unit, Shenzhen Bao’an Shiyan People’s Hospital, Shenzhen, China; 2 Department of Emergency, Shenzhen Bao’an Shiyan People’s Hospital, Shenzhen, China

**Keywords:** blunt trauma, circulatory failure, hemorrhagic shock, pleural effusion, solitary fibrous tumor

## Abstract

**Background:**

Solitary fibrous tumor (SFT) is a rare mesenchymal neoplasm primarily arising from the pleura. While the majority exhibit benign biological behavior, their clinical presentation is often indolent. Solitary fibrous tumor of the pleura (SFTP) is frequently an incidental finding during physical examinations or imaging for unrelated conditions, as patients are often asymptomatic in the early stages.

**Case presentation:**

A 37-year-old previously healthy male, a delivery driver, suffered blunt chest and abdominal trauma following an electric bike accident with right-sided chest and abdominal pain rapidly progressing to altered consciousness and circulatory failure. On arrival, he was in hemorrhagic shock (blood pressure 70/40 mmHg, heart rate 120 beats/min). Bedside ultrasonography demonstrated a large right pleural effusion. Emergency tube thoracostomy drained >1000 mL of bright red blood. and subsequent chest computed tomography revealed massive right pleural effusion and a giant heterogeneous mass (approximately 16.4 ×14.5×15.6 cm) in the right lower hemithorax adjacent to the mediastinum, with marked mediastinal shift and cardiac compression. Given ongoing shock and suspected rupture of an intrathoracic lesion, emergent right thoracotomy was performed. A pedunculated giant tumor (20×15×12cm) arising from the right diaphragmatic pleura had partially torn with active bleeding from the pedicle; the mass was completely resected. Despite aggressive resuscitation, including open pericardium and direct cardiac massage for intraoperative cardiac arrest, the patient died postoperatively from multiple organ failure following massive blood loss and prolonged low-flow time. Histopathology and immunohistochemistry (CD34+, STAT6+) supported the diagnosis of SFT.

**Conclusions:**

SFTP may remain clinically silent even when extremely large. Blunt trauma may cause catastrophic tumor vessel rupture and fatal hemothorax,accompanied by sudden circulatory failure.In unstable patients with massive hemothorax and an intrathoracic mass, rupture of a hypervascular pleural tumor including SFTP should be considered, We review the relevant literature to enhance clinical recognition and management strategies for giant SFTPs with atypical presentations.

## 1 Introduction

Solitary fibrous tumor (SFT) is a distinctive mesenchymal neoplasm of fibroblastic origin, typically presenting without symptoms (1). First described by Klemperer and Rabin in 1931 as “localized fibrous mesothelioma” (2). Although the pleura remains the classic site,SFT is now recognized to occur throughout the body, including mediastinum, lung parenchyma, meninges, kidney, and other extra-thoracic locations (3, 4). Overall incidence is extremely low (approximately 1 per 10^6 persons per year) (5). Epidemiologically,these tumors show a predilection for patients between the fifth and seventh decades of life (6).

Morphologically, SFTs are categorized by size and histological characteristics. Tumors exceeding 10 cm in diameter are often scrutinized for malignant potential, while those exceeding 15cm or occupying more than 40% of a hemithorax are classified as “Giant Solitary Fibrous Tumors of the Pleura” (Giant SFTP) (7).Giant size not only increases surgical complexity but may increase vulnerability to mechanical traction and vascular compromise, particularly in pedunculated tumors with fragile feeding vessels.

The clinical course of SFTP is typically indolent. Most tumors grow slowly and remain asymptomatic until they reach a size sufficient to cause compressive symptoms such as cough, chest pain,or dyspnea (8, 9). Here we report a rare and fulminant presentation: sudden circulatory failure due to massive hemothorax after blunt trauma,caused by partial tearing of the pedicle of a giant pleural SFT.We describe the diagnostic and therapeutic course, highlight key imaging and pathologic findings,and review relevant literature to improve recognition of this life-threatening but potentially underappreciated scenario.

## 2 Case report

### 2.1 General information

A 37-year-old man working as a food delivery courier,previously healthy and without chronic medical conditions,presented to the emergency department 2 hours after a traffic accident while riding an electric bicycle.He reported impact to the right chest and abdomen,followed by severe pain. Shortly thereafter,he developed progressive altered mental status and coma.He denied prior chest tightness,chest pain,cough, dyspnea,fatigue,weight loss,or other constitutional symptoms.He had no history of hypertension,diabetes mellitus,coronary disease,prior major trauma,or previous surgery,and he reported no drug or food allergies.

### 2.2 Admission and physical examination

Upon arrival,the patient was in a critical condition,exhibiting agitation and confusion.Vital signs indicated severe hemodynamic instability(shock):body temperature was 36.5°C,heart rate was tachycardic at 120 beats per minute (bpm),respiratory rate was 28 bpm, and blood pressure was hypotensive at 70/40 mmHg.Physical examination revealed contusion marks over the right chest wall with subcutaneous swelling and marked tenderness. There was no clear crepitus or abnormal chest wall motion. Breath sounds were markedly reduced in the right lung compared with the left, without obvious rales. Cardiac rhythm was regular without audible murmurs. The abdomen was soft and flat with right-sided tenderness and no rebound tenderness.

### 2.3 Laboratory tests

Emergency laboratory testing (Hb:140g/L, coagulation profile, renal and liver function tests and electrolytes) was reported as within normal limits on arrival. In the setting of abrupt hemorrhage, early labs may underestimate severity before equilibration and resuscitation.

### 2.4 Imaging and bedside assessment

Immediate bedside ultrasound demonstrated a large anechoic area measuring approximately 9.8 cm×9.9 cm in the right thoracic cavity with poor internal acoustic transmission,suggestive of massive complex pleural effusion or hemothorax.

**Figure 1:**
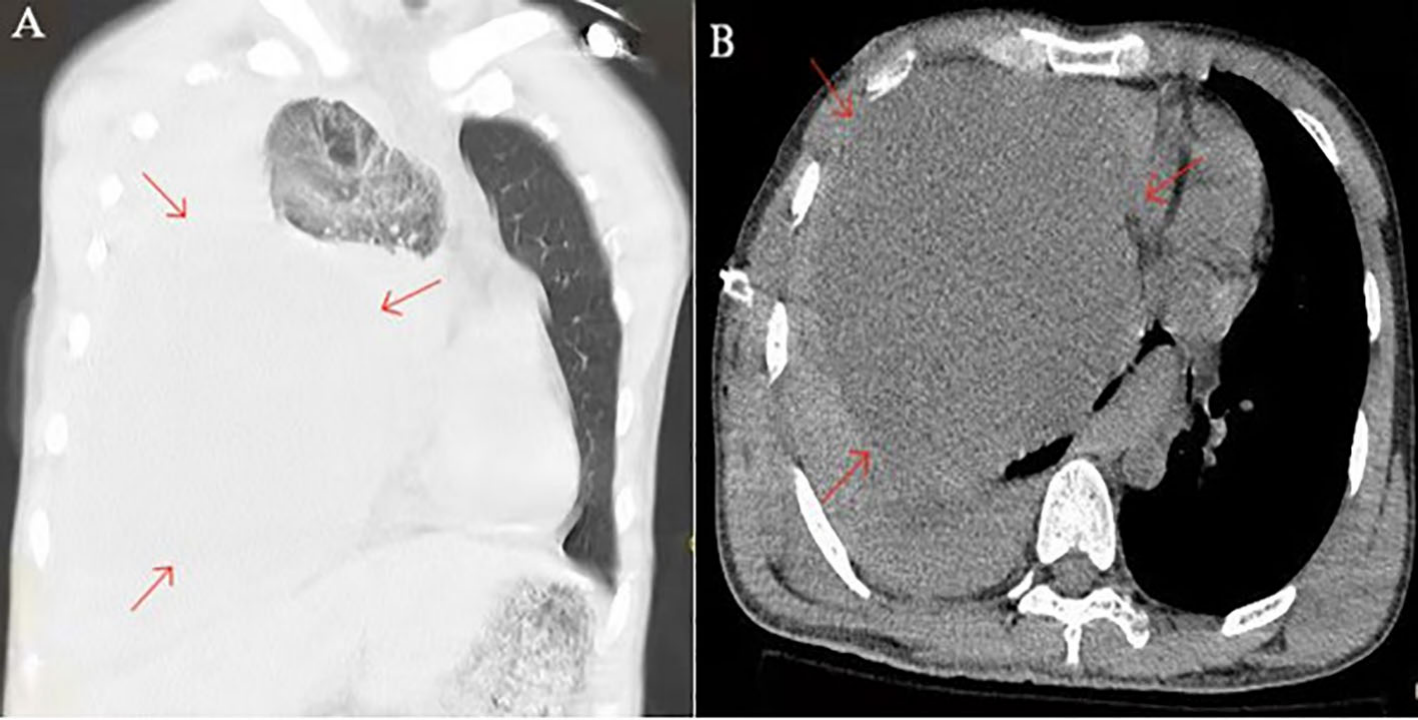
CT imaging.Coronal **(A)** and axial **(B)** images features of solitary fibroma (red arrows).

Subsequent chest computed tomography (CT) demonstrated:Massive right-sided pleural effusion;Huge space-occupying lesion in the right lower thoracic cavity adjacent to the mediastinum, approximately 16.4×14.5×15.6 cm, with heterogeneous density;Adjacent right lung compression and impaired expansion;Marked mediastinal shift to the left, resulting in compression of the cardiac chambers and contralateral displacement of the heart (**Figure 1**).

Based on the history of trauma, the signs of hemorrhagic shock, and the imaging findings, the preliminary diagnoses were traumatic hemorrhagic shock, traumatic massive hemothorax, and a right mediastinal space-occupying lesion of undetermined etiology (suspected rupture).

### 2.5 Surgical intervention and management

In view of the patient’s refractory shock despite fluid resuscitation, urgent transfer to the operating room was undertaken. After induction of anesthesia with single-lumen endotracheal intubation, thoracotomy was performed, followed by hemostasis, evacuation of the hemothorax, resection of the mediastinal tumor, and cauterization of pleural adhesions.

**Figure 2:**
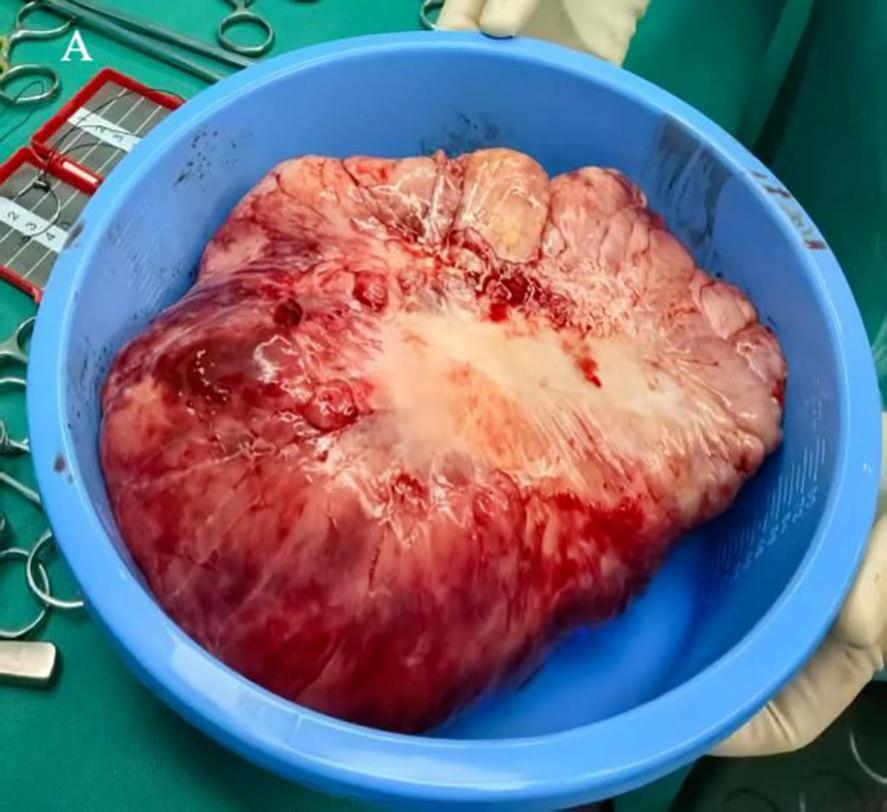
Appearance of surgically removed tumor.

Intraoperative findings:Intraoperatively, an autologous blood salvage system was established. The patient was placed in the left lateral decubitus position, and a right thoracotomy was performed via the sixth intercostal space along the midaxillary line. Approximately 2000 mL of blood and clot were evacuated from the right pleural cavity. Localized fibrous adhesions were observed between the right lower lung and the chest wall. A giant mediastinal mass, measuring approximately 20 × 20 × 15 cm, was identified in the right lower pleural cavity. The mass was pedunculated, and its pedicle originated from the right cardiophrenic region, with rupture and active hemorrhage noted at the pedicle base. Both the proximal and distal ends of the pedicle were clamped with hemostatic forceps, and the mass was subsequently completely excised (**Figure 2**).

Intraoperative laboratory monitoring revealed a hemoglobin level of 92 g/L, a lactate level of 12.4 mmol/L, and impaired coagulation function (APTT: 46.2 s; PT: 19.2 s; FIB: 0.59 g/L). At 20:35, the patient developed severe bradycardia, with the heart rate dropping to 42 beats/min. Cardiopulmonary resuscitation was immediately initiated with repeated intravenous boluses of epinephrine (1 mg each; total dose: 10 mg). To facilitate open cardiac massage, the pericardium was opened approximately 10 cm above the phrenic nerve. Following approximately 20 minutes of active resuscitation, the patient’s condition was stabilized. Total intraoperative estimated blood loss was 4500 mL, which included approximately 2000 mL from a hemothorax. Fluid and blood resuscitation included 1500 mL of salvaged autologous blood, 21 units of packed red blood cells, 1750 mL of fresh frozen plasma, 20 units of cryoprecipitate, and 1 therapeutic unit of platelets. Postoperatively, the patient remained intubated and was transferred to the intensive care unit (ICU) for further management under a continuous norepinephrine infusion (2.0 μg/kg/min).

### 2.6 Pathology and outcome

Despite maximal postoperative supportive treatment in the ICU, the patient died shortly after surgery. The death was considered to have resulted from hemorrhagic shock with subsequent cardiac arrest, systemic ischemia and hypoxia, and irreversible multiple organ dysfunction syndrome (MODS).

**Figure 3:**
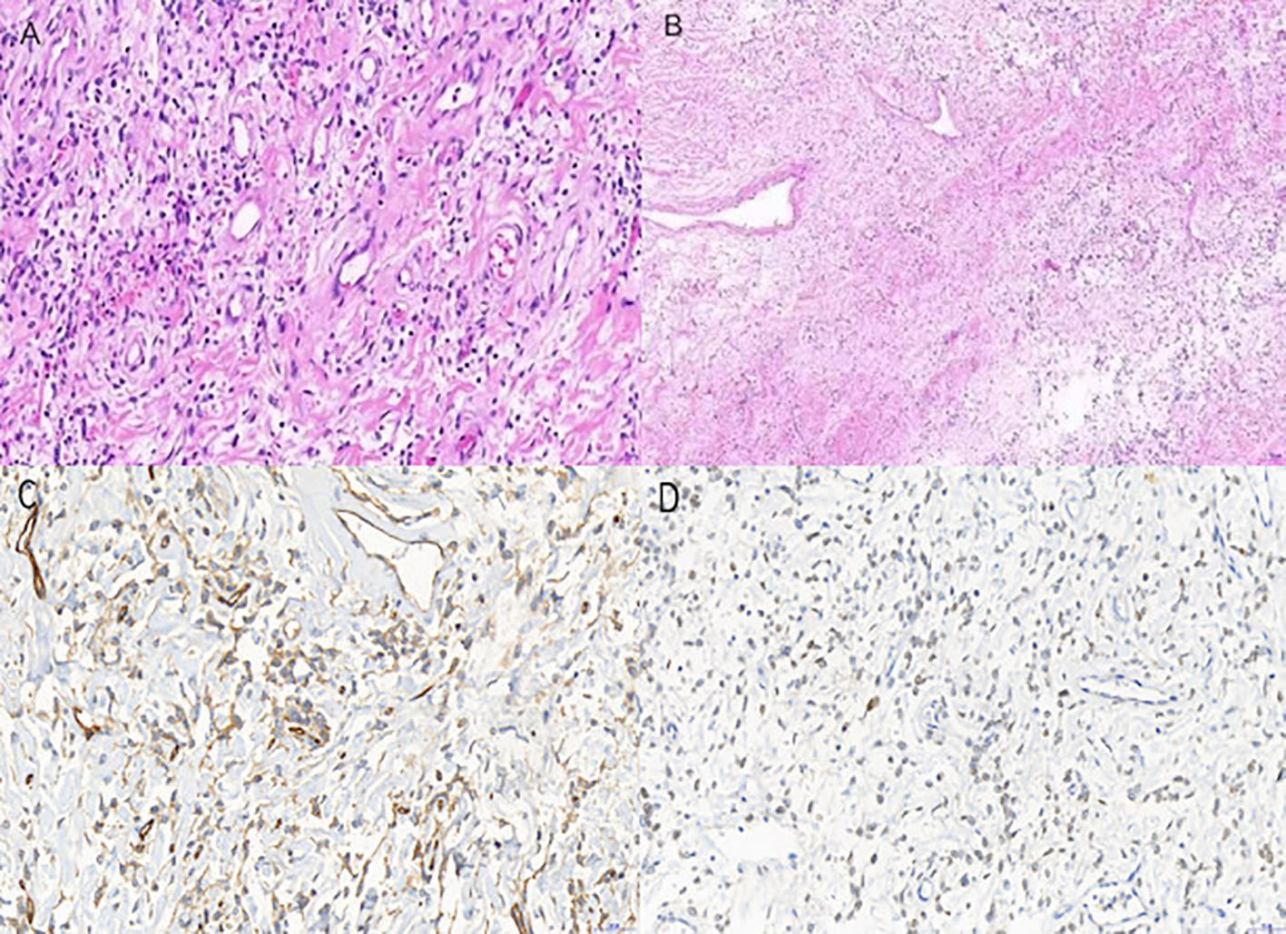
Pathological image: **(A)** HE staining; **(B)** Unique horn-like changes in blood vessels. **(C)** IHC CD34; **(D)** IHC CD34 STAT6.

Grossly,the resected specimen was a large,well-demarcated mass consistent with a mesenchymal tumor.Histopathology described a mesenchymal neoplasm. Immunohistochemical analysis supported SFT, including positivity for CD34 and STAT6. The reported diagnosis was solitary fibrous tumor (SFT) with ICD-O code 8815/1. The broader immunoprofile provided included CD34(+), CD99(+), STAT6(+), Bcl-2(+), with negative staining for CK, CDK4, CD117, S-100, and MDM2(-), and a Ki-67 index of approximately 2%, consistent with SFT (**Figure 3**).

### 2.7 Timeline of clinical course

Across the entire course of the disease, we summarized the clinical information in a chronologically ordered table (**Table 1**).

**Table 1:** Timeline of key clinical events throughout the disease course.

Time	Event
16:15	Road traffic accident
16:58	Admitted to the emergency resuscitation room; fluid resuscitation and anti-shock treatment initiated
17:10	Bedside ultrasonography revealed massive right pleural effusion
17:36	Closed thoracic drainage performed; 1200 mL of hemorrhagic fluid drained
17:53	Transfusion initiated: 20 U packed red blood cells and 800 mL plasma
18:04	Chest CT showed a giant space-occupying lesion in the right thoracic cavity
18:13	Central venous catheter placed
18:17	Endotracheal intubation performed
18:25	Transferred to the operating room
20:35	Cardiac arrest; CPR initiated
22:15	Surgery completed
22:20	Transferred to the ICU; ongoing resuscitation, norepinephrine infusion, and mechanical ventilation
00:34 (next day)	Death

## 3 Discussion

### 3.1 Epidemiology

Solitary fibrous tumors are rare neoplasms accounting for less than 5% of all pleural tumors. While Klemperer and Rabin established the pleural origin in 1931 (2), subsequent research has demonstrated that SFTs are ubiquitous mesenchymal tumors that can arise in extrathoracic sites including the meninges, soft tissues, and solid organs (3, 4). The annual incidence is low, estimated at 1 per million (5). A distinct feature of the case presented is the patient’s age. SFTs typically present in the fifth to seventh decades of life (6). Our patient, at 37 years old, represents a younger demographic cohort, which is less commonly described in the literature.

### 3.2 Clinical presentation: from silent to catastrophic

The clinical spectrum of SFTP is broad and largely dictated by tumor size.Small tumors are almost invariably asymptomatic and are discovered incidentally during radiographic screening for other conditions.As the tumor grows,symptoms arise from mass effect.The most common presenting complaints described in the literature are chest pain,cough,and dyspnea (8, 9).

The correlation between tumor size and symptomatology has reported in the literature. Wang et al. (9) and de Perrot et al. (10) noted that chest symptoms are frequent when the tumor diameter exceeds 10 cm. Furthermore, Chu et al. reported that tumors larger than 20 cm are almost universally symptomatic due to severe compression of the lung and mediastinum (11).

In contrast to the typical indolent course,our patient presented with abrupt hemorrhagic shock and circulatory collapse after blunt trauma. Despite harboring a “Giant” SFTP (20 cm diameter), he remained asymptomatic and functionally active as a delivery worker until the moment of trauma. This implies a slow growth rate allowing for physiological compensation.The sudden transition from an asymptomatic state to catastrophic circulatory failure highlights a precarious vulnerability of giant SFTs: their vascular supply. England DM et al. noted that two-thirds of these tumors attach to the visceral pleura via a pedicle (12). Similarly, surgical exploration in our patient revealed a pedunculated tumor arising from the right diaphragmatic pleura.In this patient, a large pedunculated intrathoracic fibrous tumor was present within the thoracic cavity. During high-speed travel on an electric motorcycle, the patient sustained a severe impact. Inertial forces likely displaced the tumor relative to its original position, producing marked traction on the pedicle. This traction plausibly caused tearing of vessels at the pedicle base, resulting in acute intrathoracic hemorrhage. Given the relatively rich vascularity of such tumors, rapid and massive blood loss may occur within a short period, culminating in hemorrhagic shock. This mechanism transforms a benign neoplasm into a lethal hemorrhagic event,a presentation that is exceptionally rare in the existing literature.

### 3.2 Imaging characteristics

Chest radiography is an important initial diagnostic modality, and its appearance varies with tumor size. Solitary fibrous tumors (SFTs) typically manifest as round or lobulated, well-circumscribed masses, with or without an associated pleural effusion (13). However, the limited soft-tissue contrast of plain radiography often necessitates further evaluation.Chest radiography is an important initial diagnostic modality, and its chest computed tomography (CT), with its superior soft-tissue resolution, more accurately delineates the relationship between the tumor and the pleura, which is crucial for precise localization, treatment planning, and prognostic assessment. Small SFTs usually appear as well-defined, homogeneous soft-tissue nodules adjacent to the chest wall, forming an obtuse angle with the pleural surface. In contrast, larger lesions tend to exhibit heterogeneous attenuation and form an acute angle with the pleura, thereby mimicking subpleural pulmonary masses and predisposing to misdiagnosis as peripheral lung cancer (14).

In the present case, the patient was admitted after trauma and had no prior imaging examinations. Emergency CT revealed a large right-sided pleural effusion. A huge, space-occupying lesion with heterogeneous density was identified in the right inferior mediastinum, measuring approximately 16.4 cm×14.5cm×15.6cm. The mass compressed the adjacent right lung, resulting in atelectasis and leftward displacement of the mediastinum and heart. These findings are consistent with the CT characteristics of a large SFT with malignant potential. Notably, this case is distinctive in that the patient presented with massive hemothorax caused by rupture of a vessel within the tumor pedicle, a complication that has been only rarely documented in the existing literature.

### 3.3 Pathology and immunohistochemistry

Pathological examination is fundamental for establishing a definitive diagnosis of solitary fibrous tumor (SFT).Accurate diagnosis relies on an integrated assessment of histopathological features in combination with immunohistochemical profiling. CD34, CD99, STAT6, and Bcl-2 are currently regarded as the most sensitive and specific immunohistochemical markers for SFT (15).

In this patient, immunohistochemical analysis of the postoperative specimen demonstrated positivity for CD34, CD99, STAT6, and Bcl-2, and negativity for CK, CDK4, CD117, S-100, and MDM2. The Ki-67 labeling index was approximately 2%,indicating low proliferative activity.Collectively, these findings are consistent with,and fulfill the immunophenotypic criteria for,a diagnosis of solitary fibrous tumor.Previous research by Zhang et al. indicates that the Ki-67 proliferation index for benign solitary fibrous tumors (SFTs) typically ranges from 1% to 10%, whereas malignant variants exhibit indices between 5% and 70% (16). In the present case, the patient’s Ki-67 labeling index was approximately 2%, confirming low proliferative activity and categorizing the lesion as histologically benign. However, this case underscores a critical clinico-pathological discrepancy: a favorable immunohistochemical profile does not preclude a catastrophic clinical outcome. In giant pedunculated thoracic tumors, the vascularized pedicle is highly vulnerable to significant traction, torsion, or shear forces—particularly during blunt trauma or rapid deceleration. Such mechanical stress can precipitate the rupture or avulsion of the pedicle base, leading to massive hemothorax. Consequently, emergency physicians and surgeons must recognize that histologically low-grade tumors may still pose a risk of fatal vascular events, independent of oncologic invasiveness.

### 3.4 Treatment

Surgical resection remains the mainstay of treatment for SFT, and patient prognosis is closely related to the completeness of tumor removal and the presence or absence of histological malignancy (17). The clinical course in the present case was fulminant. Blunt trauma led to rupture of vessels within the tumor pedicle,causing acute massive intrathoracic hemorrhage and resulting in a large hemothorax. To control the bleeding and attempt to save the patient’s life, an emergency thoracotomy was performed, during which the SFT was completely excised and hemostasis was achieved.Despite the complete resection of the tumor, massive preoperative and intraoperative blood loss precipitated severe hemorrhagic shock and cardiac arrest. Consequently, the patient suffered prolonged systemic ischemia and hypoxia, ultimately succumbing to irreversible multiple organ dysfunction syndrome (MODS).

We searched the PubMed database for English-language literature published between 2000 and April 2026, using the keywords ‘solitary fibrous tumor’ and ‘hemothorax’. The initial search yielded 11 articles. After rigorous screening, 5 articles that met the inclusion criteria were included in this review, representing a total of 5 patients. The relevant clinical characteristics of these cases are summarized in **Table 2**.

**Table 2:** Five cases of giant solitary fibrous tumor reported in the literature (18–22).

Author	Year	Age	Tumor size(cm)	Presentation	Trigger	Management	Outcome
Asai K et al. (18)	2003	31	9	chest pain and dyspnea	surfing	Surgical resection	survival
Tekinbas C et al. (19)	2008	45	23	none	fall	Surgical resection	survival
Negri G et al. (20)	2014	38	7	chest pain and dyspnea	spontaneous	Surgical resection	survival
Tan JH et al. (21)	2016	76	9.7	Haemoptysis chest pain hoarseness of voice	spontaneous	Surgical resection	survival
Komatsu H et al. (22)	2024	48	5	chest pain	spontaneous	Surgical resection	survival

Previous literature suggests that spontaneous or trauma-induced hemorrhage and hemothorax associated with solitary fibrous tumor of the pleura (SFTP) are rare but clinically critical events that require heightened vigilance. Asai and Tekinbas et al. reported acute symptom onset following surfing and a fall, respectively, indicating that tumor-related hemothorax may be precipitated even by low- to moderate-energy external forces (18, 19). In contrast, Negri, Tan, and Komatsu reported cases without a clear history of trauma; clinical manifestations included chest pain, dyspnea, hemoptysis, and hoarseness, suggesting that tumor-related bleeding may also occur at rest (20–22). Notably, a shared feature of these reports is that emergency surgery achieved definitive hemorrhage control and tumor resection, resulting in survival.

In contrast to these successfully treated cases, the present case resulted in a fatal outcome. The patient developed progressive massive bleeding and rapidly progressed to hemorrhagic shock soon after high-energy trauma, supporting the hypothesis that external forces may cause inertial displacement and traction of a pedunculated tumor, leading to tearing or avulsion of the pedicle vessels and subsequent massive hemothorax. Therefore, clinical risk assessment in SFTP should not rely solely on histologic grade or immunophenotype; it should also incorporate tumor burden, pedunculated anatomy, and exposure to high-energy trauma. This case further suggests that in patients with high-energy chest trauma and an intrathoracic mass, early risk stratification and prompt hemorrhage-control strategies should be prioritized to reduce the risk of catastrophic vascular events.

### 3.5 Limitation

Several limitations of this report warrant consideration.therefore, the observed clinical manifestations and the proposed injury mechanism may not be generalizable to all patients with large solitary fibrous tumors of the pleura (SFTP).Consequently, it is difficult to estimate the true incidence of trauma-induced pedicle rupture and hemorrhage within the broader SFT population.Second, the absence of pre-traumatic imaging precludes a definitive assessment of the tumor’s baseline anatomical orientation, pedicle length, and specific attachment site. This lack of prior data also makes it impossible to determine whether the basal vasculature was under pre-existing tension or if events, such as localized micro-hemorrhages, had occurred before the impact.Finally, the patient’s fulminant presentation necessitated immediate life-saving resuscitation and surgical intervention, which resulted in fragmented laboratory data.Specifically, the lack of serial monitoring for hemoglobin levels, lactate clearance, and coagulation profiles.While indicative of the practical difficulties encountered during life-saving interventions, these limitations hinder the objective quantification of hemorrhage severity and its associated compensatory mechanisms.

## 4 Conclusion

This case report describes a rare and fatal presentation of a giant solitary fibrous tumor of the pleura (SFTP) in a young male patient. Although SFTs are generally indolent, this case demonstrates that giant lesions may remain clinically silent until precipitated by traumatic injury. Beyond oncologic considerations, this report highlights vascular catastrophe as a rare but potentially lethal mode of presentation in giant pedunculated tumors.Recognition of this mechanism is critical for emergency physicians and trauma surgeons.

Based on the insights from this case, we propose the following recommendations for trauma teams managing patients with chest trauma in the presence of an intrathoracic tumor.In patients presenting with high-energy blunt trauma and imaging studies revealing or strongly suggesting an intrathoracic mass, the tumor itself must be considered a primary source of bleeding. Hemodynamic deterioration disproportionate to the external signs of injury, or a rapidly accumulating hemothorax in the absence of obvious osseous or parenchymal injury, should raise strong suspicion for hemorrhage from pedicle avulsion or tumor rupture.For hemodynamically stable or transiently responsive patients, early contrast-enhanced chest CT Angiography (CTA) is the diagnostic modality of choice. CTA is crucial not only for assessing standard traumatic injuries but also for characterizing the tumor’s morphology (size, pedicle structure, and attachment site). The detection of active contrast extravasation confirms the bleeding source and informs the decision between endovascular and surgical management.Emergency thoracotomy remains the definitive intervention for hemorrhage control in hemodynamically unstable patients or those with a massive hemothorax. This procedure should not be delayed for further diagnostic workup, as it provides the only means for direct hemorrhage control.The combination of a large intrathoracic mass and evidence of massive hemothorax should trigger preemptive activation of the MTP. This early, balanced resuscitation is critical to prevent or reverse trauma-induced coagulopathy (TIC) and to support perfusion until definitive hemorrhage control is achieved.

## Data Availability

The original contributions presented in the study are included in the article/supplementary material. Further inquiries can be directed to the corresponding author.
